# Illicit Drug Use and Associated Problems in the Nightlife Scene: A Potential Setting for Prevention

**DOI:** 10.3390/ijerph18094789

**Published:** 2021-04-30

**Authors:** Kristin Feltmann, Tobias H. Elgán, Anna K. Strandberg, Pia Kvillemo, Nitya Jayaram-Lindström, Meryem Grabski, Jon Waldron, Tom Freeman, Helen Valerie Curran, Johanna Gripenberg

**Affiliations:** 1 STAD, Stockholm Prevents Alcohol and Drug Problems, SE-113 64 Stockholm, Sweden; tobias.elgan@ki.se (T.H.E.); Anna.Strandberg@ki.se (A.K.S.); pia.kvillemo@ki.se (P.K.); johanna.gripenberg@ki.se (J.G.); 2 Centre for Psychiatry Research, Department of Clinical Neuroscience, Karolinska Institutet & Stockholm Health Care Services, Region Stockholm, Norra Stationsgatan 69, SE-113 64 Stockholm, Sweden; nitya.jayaram@ki.se; 3 Clinical Psychopharmacology Unit, University College London, 1-19 Torrington Place, London WC1E 7HB, UK; m.grabski@ucl.ac.uk (M.G.); jonathan.waldron.15@ucl.ac.uk (J.W.); T.P.Freeman@bath.ac.uk (T.F.); v.curran@ucl.ac.uk (H.V.C.); 4 Addiction and Mental Health Group (AIM), Department of Psychology, University of Bath, Bath BA2 7AY, UK

**Keywords:** illicit drugs, abuse, nightclubs, festivals, DUDIT, anxiety, prevention, substance use disorder, gender, age

## Abstract

Illicit drug use is prevalent in the nightlife scene, especially at electronic dance music (EDM) events. The aim of the present study was to investigate illicit drug use patterns and consequences of drug use among frequent visitors of EDM events. Young adults (18–34 years old) who had visited at least six EDM events in Sweden during the past year participated in a web-based survey on drug use patterns and its consequences. Fifty-nine percent of participants had used illicit drugs during the past year, most often cannabis followed by ecstasy, cocaine, and amphetamine. Nightlife venues were identified as the main setting for the use of central stimulants, while cannabis was mostly used at home. Frequent alcohol and tobacco use was associated with illicit drug use. The most prevalent negative consequences of drug use were related to mental health, such as impairments in mood, sleep, and memory problems, but physical manifestations were also reported, such as palpitations and collapsing. These findings confirm that drug use is prevalent and associated with negative health effects among EDM nightlife attendees. The nightlife scene is a setting with promising potential to reach a high-risk target group with illicit drug use prevention interventions.

## 1. Introduction

Illicit drug use has been associated with a number of risky behaviors and acute health consequences, such as injuries, infarction, psychosis, violence, and sexual risk taking [1,2,3]. Furthermore, long-term use of illicit drugs can negatively affect physical and psychological health, leading to chronic sleep disturbances, mood disorders, cognitive impairments, and/or substance use disorders [1,4,5,6,7,8]. Considering these detrimental effects, efforts to prevent drug use in certain settings, such as different nightlife settings, might mitigate these risks. More knowledge on groups who use illicit drugs, settings where drugs are used, as well as characteristics of patterns of use, is needed to inform the development of preventative interventions. 

The European Monitoring Centre for Drugs and Drug Addiction (EMCDDA) reports that illicit drug use is highest among young adults [9]. In Sweden, the percentage of 17- to 34-year-olds who have used drugs in the past year was 10% for cannabis, 3% for cocaine, 2% for ecstasy (3,4‑Methylenedioxymethamphetamine: MDMA), and 1% for amphetamine [10]. These figures are similar to the European averages with the exception of cannabis, which is under the European average of 15% [9]. Previous research shows that drug use is common among attendees of nightclubs, bars, pubs, and festivals and particularly at electronic dance music (EDM) events [4,11,12,13,14,15,16]. In the Nordic countries, studies on drug use in the nightlife scene show a higher drug use prevalence among nightlife attendees than among the general population [14,17,18,19,20,21,22,23]. For example, studies at Norwegian clubs and festivals show that 43% and 21%, respectively, of the attendees had used illicit drugs during the previous year [20,23]. Furthermore, 25% of club goers in Norway tested positive for using an illicit drug [21]. In a Swedish study at an EDM event on a cruise ship, 9% of attendees reported that they usually use illicit drugs when clubbing and 10% tested positive for at least one illicit drug [18]. 

The most common illicit drugs used by nightlife attendees are cannabis, ecstasy, cocaine, and amphetamine [13,15,16,17,20,23,24,25,26,27]. The use of cannabis has been associated with an increased risk of psychosis, including hallucinations and delusions, and also acute anxiety (e.g., panic attacks) [1,28]. Ecstasy use has also been associated with an increased risk of overheating, sweating, and dehydration, especially while dancing [29]. Consequently, moving to colder environments and drinking excessive amounts of fluid can then lead to hypothermia or hyponatremia (i.e., decreased blood sodium levels). Due to these effects and, in the past decade, a rise in the average MDMA content but also the presence of toxic adulterants such as para-Methoxyamphetamine (PMA) in ecstasy pills, ecstasy use has led to fatalities [29,30,31]. Cocaine and amphetamine use has also been associated with an increased risk of psychotic symptoms and myocardial infarction, which in turn can trigger fatal cardiac arrhythmias and stroke [1,32]. In addition, regular use of cannabis and the abovementioned three central stimulants is associated with mood fluctuations, depression, anxiety, sleep disturbances, and cognitive deficits [5,29,33,34,35,36]. 

There are a substantial proportion of drug users who use several drugs during the same occasion or within a given window of time (polydrug use) [4,15,37,38,39]. Several studies show that many drug users drink alcohol while using illicit drugs [18,21,23,40,41,42,43,44] and that hazardous drinking is associated with illicit drug use [18,20,40,42]. Polydrug use is associated with mental health problems, sexual risk taking, and violence [3,6,33,45]. Most of the abovementioned negative consequences have been described among samples of recreational drug users in general [5,34,46,47,48,49,50], and few studies have investigated the extent to which nightlife goers experience adverse consequences related to their drug use [4,44,45,51]. For example, a recent study among 1092 attendees of EDM events in New York showed that one-third reported to have experienced adverse effects related to drug use during the past year. Alcohol was most commonly mentioned as causing adverse outcomes, followed by cannabis [44]. In another study, using a web-based survey, a considerable number of nightlife goers reported having experienced negative consequences, such as bad mood, hangover, insomnia, memory problems, and tachycardia, especially among the heavy polydrug users [45,51]. Another study at two EDM events in Switzerland showed that party drug use was strongly associated with depressed mood, sleeping problems, and anxiety attacks, as well as accidents and emergency treatment [4]. 

Apart from the types of substances used, there is research on which factors influence illicit drug use. While some studies show that illicit drug use and polydrug use is more prevalent among males than females [13,40,52], other studies, including two Swedish nightlife studies, show small or no gender differences [4,17,18,53]. Furthermore, nightlife studies have shown a higher prevalence of illicit drug use among homo- and bisexual persons compared with heterosexual individuals [54,55]. In contrast, in another study, being male and heterosexual was related to the use of cannabis in these settings [40]. Studies among Swedish nightlife staff and Belgian nightlife goers have shown that illicit drug use was most prevalent among the youngest age groups of young adults [17,52,56].

Despite studies demonstrating drug use in the nightlife scene and studies associating negative consequences with drug use, in-depth knowledge of drug use and related problems is scarce among nightlife settings internationally, as well as in Sweden. The aim of the present study was to investigate illicit drug use among Swedish young adults regularly attending EDM nightlife events. More specifically, we wanted to identify the prevalence and patterns of drug use and factors influencing drug use and to investigate negative consequences after using drugs. Findings of the present study demonstrate the need for a preventive intervention in nightlife venues and could help guide the development of such an intervention.

## 2. Materials and Methods

### 2.1. Participants and Procedure

The present study is part of the ALAMA nightlife study, including five European countries: the Netherlands, the UK, Belgium, Italy, and Sweden. In the period May to November 2017, an online survey was conducted among young adults (age 18–34) frequently visiting EDM events (at least six times during the past 12 months). In the present study, data from Sweden were analyzed.

Participants were recruited through paid, targeted advertising on the Facebook and Instagram social media platforms. The following targeting criteria were selected for the adverts: a range of popular nightclubs, DJs, music genres, events, and news groups in each country. Furthermore, online groups, forums, and websites focusing on electronic dance music were contacted to advertise the survey (see Supplementary Table S1). Inclusion criteria were: age 18–34, attended at least six electronic dance music events in the past 12 months, and residing in Sweden. The exclusion criterion was stating the use of the fictional drug ‘spanglers’. The age range was chosen to match the upper age limit of the EMCDDA’s definition of a ‘young adult’, while the number of events was chosen to ensure sufficient engagement with the European nightlife scene. The survey contained questions on demographics, nightlife (music and venues), illicit drug use, general well-being (WHO1-5), risk perceptions, and experiences of negative and positive consequences and harm reduction methods. Illicit drugs were defined as substances classed as narcotics in Sweden, which are psychoactive compounds with abuse potential, including both recreational and prescription drugs. The study was approved by the Regional Ethical Review Board in Stockholm (Dnr 2017/977-31/5). 

### 2.2. Statistical Analysis

Participants who fulfilled the inclusion criteria, did not fulfil the exclusion criterion, and had completed the survey were included in analysis. Data on demographics and drug use were analyzed and presented in frequencies (%) and number of participants, means, and standard deviations (SD), as well as medians and interquartile ranges for skewed distributions. Whereas ever and past 12-month use was presented as a percentage of all participants (no dropouts), frequency of drug use was presented as a percentage of drug users, i.e., those who had stated the use of the specific drug during some point in their life (ever users) and were therefore shown the question on frequency. When analyzing drug use patterns, only illicit drugs with a 12-month use prevalence above 10% were included to ensure enough data for each drug.

Drug use in settings and consequences of drug use are presented as a percentage of respondents to each question due to varying degrees of dropout. Pearson’s chi-squared tests were performed to explore possible differences in illicit drug use related to age and/or gender. For the analysis regarding differences in age of onset of different drugs (repeated measures), the Friedman test was used due to a non-normal distribution of the standardized residuals. Furthermore, to explore the possible correlation between number of drugs used during the last 12 months (overdispersed data) and the frequency of alcohol or tobacco use, a negative binomial regression was used. A binary logistic regression was used to explore potential factors associated with illicit drug use during the last 12 months (for inclusion of factors, see Appendix A). The medians of the composite score of the general well-being questionnaire (WHO1-5) were compared between drug use categories, as assessed with the DUDIT-1 question using the Kruskal–Wallis test. 

### 2.3. Dropout Analysis

To investigate if the sample included in the analyses was representative of all participants who started the survey, characteristics of excluded participants were analyzed (see, Supplementary Table S2). The participants excluded from analyses were divided into two groups: those who answered the items on illicit drug use (*n* = 625) and those who had stopped the survey before completing it (*n* = 1002). 

Distributions of age and gender were similar between participants included and excluded from the analyses (Table S2). The prevalence of lifetime use of cannabis, ecstasy, cocaine, and amphetamine was higher among participants who had answered questions on drug use but did not complete the survey or fulfil the inclusion criteria than among participants included in analysis. However, last 12-month use was lower among excluded compared with included participants for cannabis, ecstasy, cocaine, and amphetamine. It should be noted that a large proportion of illicit drug users chose not to answer the question of 12-month use, whereas there was no internal drop-out on this question among the survey completers included in the analyses.

## 3. Results

### 3.1. The Study Population: Demographics and Attendance at Nightlife Events

In total, data from 1371 respondents were analyzed. Participants had a mean age of 24.8 (SD: 4.5), and the majority were male, heterosexual, had completed high school, were living in a large town/or city, and were single. Table 1 displays the distribution frequencies of the demographic characteristics. 

The median number of EDM events visited during the past 12 months were 15 (interquartile range: 10–25). The most common music styles played at the events were house and techno, followed by electro, hip-hop, RnB, trance, and disco (see, Supplementary Table S3). The most important motives to go out were ‘to have fun’, ‘to listen to music’, and ‘to dance’, followed by ‘because my friends are going’ and ‘to see a particular artist’ (see Supplementary Table S4). Whereas 13.1% and 1.6% rated ‘to take drugs’ as ‘slightly’ or ‘very important’, respectively, 57.0% rated this motive as not important (Table S4). Whereas most participants (over 90%) had ever been to clubs, legal festivals, house parties and pubs, fewer (67.7%) had ever been to an illegal festival (see, Supplementary Table S5).

### 3.2. Prevalence of Drug Use 

**Figure 1:**
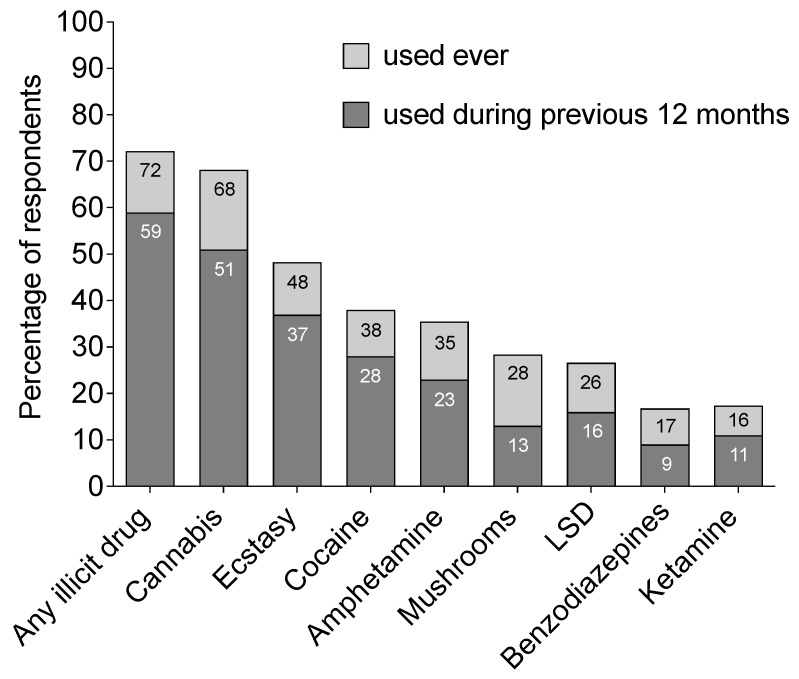
Drug use prevalence: lifetime and use during the past 12 months. Participants stated for each drug if they had used the drug during their lifetime (light grey) or during the last 12 months (dark grey). Within each bar, the percentage of people having used the drug is given. There are no missing data. However, participants stating no lifetime use for a certain drug were not shown the question on use of this drug during the last 12 months. Data are presented as percentage of the whole sample (*n* = 1371). Illicit drugs are defined as substances classed as narcotics in Sweden, which are psychoactive compounds with abuse potential, including both recreational and prescription drugs. Excluded from this category were alcohol and tobacco and nitrous oxide or amyl/alkyl nitrates (poppers) since they are not classed as narcotics in Sweden. LSD, lysergic acid diethylamide.

Whereas 71.9% of the participants had used illicit drugs at some point during their life, 58.7% had been using illicit drugs during the past 12 months (Figure 1). A majority reported ever use of cannabis (68.2%) and almost half reported ever use of ecstasy (47.8%). More than one in three participants had ever used cocaine (37.9%) or amphetamine (35.4%). The use of other drugs, such as novel psychoactive substances (NPS), was less prevalent (Table 2). Regarding drug use during the last 12 months, about one in two (51.0%) and one in three (36.5%) had been using cannabis and ecstasy, respectively (Figure 1). Almost all (97.2%) of the respondents had used alcohol during their lifetime and 94.7% had used alcohol during the last 12 months. Tobacco products (cigarettes, moist snuff/snus, etc.) were used by 82.5% during their lifetime and by 71.3% during the last 12 months.

### 3.3. Setting and Frequency of Drug Use

Most participants reported that the main setting for illicit drug use was their own or a friend’s home for the use of cannabis (65.0%), mushrooms (49.7%), or lysergic acid diethylamide (LSD, 60.3%). Most participants also reported illegal festivals as the main setting for ecstasy (35.1%), amphetamine (29.6%), and ketamine use (24.2%). Night clubs were reported as the most common and second most common setting for cocaine (38.9%) and amphetamine use (17.4%), respectively (Table 3). 

**Figure 2:**
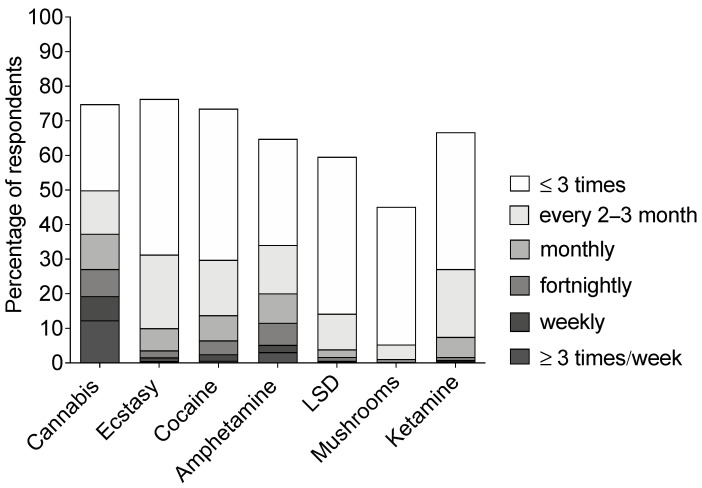
Drug use frequency of drug users. Frequency of drug use during the past 12 months is presented as percentage of ever users for each drug. Data are stacked as to show ‘at least’ drug use frequency. For example, whereas almost 20% (of people who had ever used cannabis) reported using cannabis at least weekly, around 50% reported using cannabis at least every 2–3 months. LSD, lysergic acid diethylamide.

For most drugs, the majority of drug users had not been using the drug or had been using the drug between one and three times during the past 12 months (Figure 2). For cannabis, about half of the users (49.8%) had used cannabis at least every two to three months, 37.2% had used it at least monthly, 19.3% used it at least weekly, and 12.3% used it at least three times a week during the past 12 months. About one in three (34.0%) had used amphetamine at least every two to three months. Almost equally often were the following drugs used (at least every two to three months): ecstasy 31.3%; cocaine 29.9%; and ketamine 27.1%. Whereas 20.0% of amphetamine users had used amphetamine at least monthly during the past 12 months, the corresponding figures were 13.9% for cocaine, 9.9% for ecstasy, and 7.6% for ketamine (Figure 2). In comparison, almost half of the alcohol users (48.6%) used alcohol at least weekly, and 86.8% used alcohol at least monthly during the past 12 months. Tobacco products (cigarettes, moist snuff/snus, etc.) were used at least weekly by 55.5% of users and at least monthly by 69.5% of users during the past 12 months. 

### 3.4. Factors Associated with Illicit Drug Use

#### 3.4.1. Age and Gender

**Figure 3:**
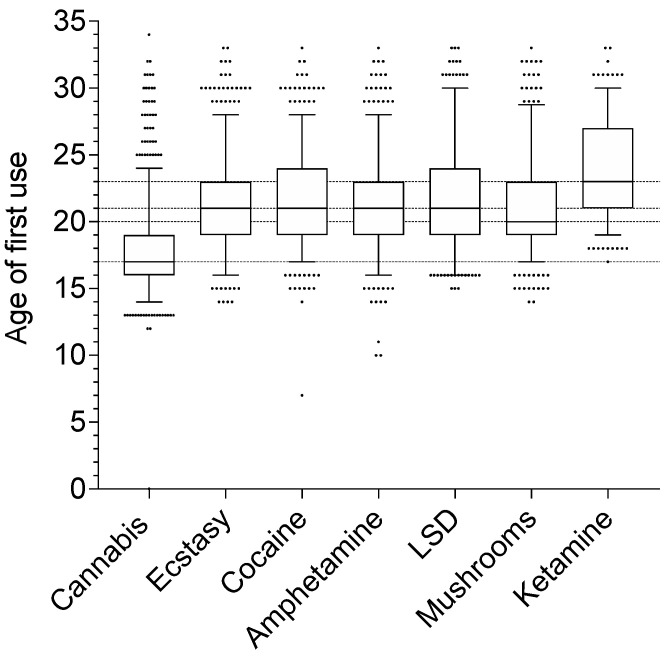
Age of first use of each drug. The median age of first use is shown (line) for each drug. The box shows the 25th and 75th percentiles and the whiskers show the 5th percentile. The remaining data points are shown as individual points stacked next to each other. LSD, lysergic acid diethylamide.

Regarding illicit drugs, the median age of onset was significantly different between the different drugs (Friedman’s Q = 334.5, *p* < 0.001). The age of onset was lowest for cannabis (median: 17; interquartile range (IQR): 16–19) and highest for ketamine (median: 23; IQR: 21–27) (Figure 3). The median age of onset was 20 for magic mushrooms (IQR: 19–23) and 21 for ecstasy (IQR: 19–23), amphetamine (IQR: 19–23), cocaine (IQR: 19–24), and LSD (IQR: 19–24). As a comparison, the median age for first use was 15 for alcohol (IQR: 14–16) and 16 for tobacco products (IQR: 14–17), respectively.

**Figure 4:**
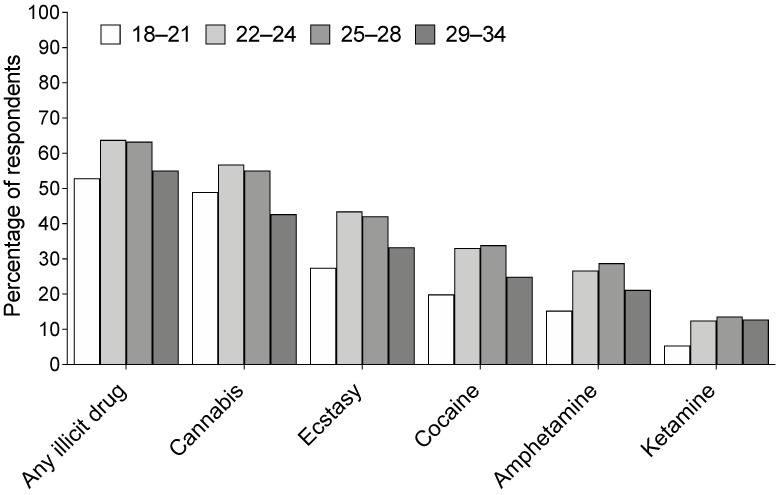
Drug use during the last 12 months depending on age group. Drug use prevalence during the last 12 months was significantly different between age groups for the drugs displayed (chi-square). In contrast, use of LSD (lysergic acid diethylamide) and hallucinogenic mushrooms did not differ between age groups. Drug use prevalence is presented as percentage of all respondents (*n* = 1371).

Use of illicit drugs during the past 12 months was more prevalent among age groups 22–24 and 25–28 years than among age groups 18–21 and 29–34 (X^2^ = 13.47 (3), *p* = 0.004, Figure 4). Use during the past 12 months differed significantly between age groups for cannabis (X^2^ = 16.31 (3), *p* = 0.001), ecstasy (X^2^ = 25.83 (3), *p* < 0.001), cocaine (X^2^ = 23.94 (3), *p* < 0.001), amphetamine (X^2^ = 22.44 (3), *p* < 0.001), and ketamine (X^2^ = 15.74 (3), *p* = 0.001). Ketamine use was most frequent in older age groups, while the remaining drugs were most frequent in the two middle age groups (22–24 and 25–28 years; see Figure 4). Whereas alcohol use during the past 12 months did not differ significantly between age groups (X^2^ = 3.11 (3), *p* = 0.376), tobacco use during the past 12 months did (X^2^ = 8.29 (3), *p* = 0.040) and was 73.6% among 18- to 21-year-olds, 74.8% among 22- to 24-year-olds, 71.2% among 25- to 28-year-olds, and 65.4% among 29- to 34-year-olds.

Overall use of illicit drugs during the past 12 months was higher among women than men (64.8 vs. 56.4%, Table 4). Among women, a significantly higher proportion reported use of ecstasy, cocaine, and amphetamine during the past 12 months compared with men. No significant gender differences were found for the remaining illicit drugs or for alcohol (95.5% among men vs. 94.1% among women, X^2^ = 1.06 (1), *p* = 0.303) or tobacco products (70.6% among men vs. 73.2% among women, X^2^ = 0.87 (1), *p* = 0.352).

#### 3.4.2. Frequency of Use of Alcohol and Tobacco

**Figure 5:**
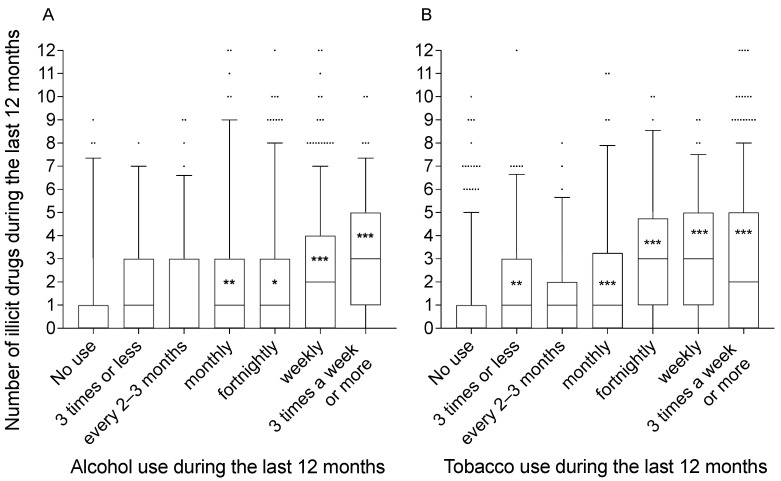
Number of illicit drugs used correlates with frequent alcohol or tobacco use. The number of illicit drugs used during the last 12 months is presented for each category of frequency of alcohol (**A**) or tobacco (**B**) use during the last 12 months. Frequency of alcohol or tobacco are predictors for the number of illicit drugs used (negative binomial regression). * *p* < 0.05, ** *p* < 0.01, *** *p* < 0.001: significant increase in the incidence rates of the number of illicit drugs used compared with the reference category ‘not used in the past 12 months’.

Negative binomial regressions revealed that the frequency of alcohol (X^2^ = 28.51 (6), *p* < 0.001) and tobacco (X^2^ = 148.04 (6), *p* < 0.001) use during the last 12 months was associated with the number of illicit drugs used during the same period (Figure 5). For alcohol, the incidence rate for the category ‘monthly’ was 1.8 times (*p* = 0.005) the incidence rate for the reference category ‘not during the last 12 months’. The corresponding factors for the remaining (significant) categories were: 1.5 times for ‘fortnightly’ (*p* = 0.025); 1.9 times for ‘weekly’ (*p* < 0.001); and 2.5 times for ‘three times a week or more’ (*p* < 0.001).

For tobacco, the incidence rates for the category ‘monthly’ was 2.2 times (*p* < 0.001) the incidence rates for the reference category ‘not during the last 12 months’. The corresponding factors for the remaining (significant) categories were: 1.5 times for ‘three times a year or less’ (*p* = 0.004); 2.8 times for ‘fortnightly’ (*p* < 0.001); 2.9 times for ‘weekly’ (*p* < 0.001); and 2.8 times for ‘three times a week or more’ (*p* < 0.001). 

#### 3.4.3. Factors Associated with Illicit Drug Use

The logistic regression model was statistically significant (X^2^(25) = 393.11, *p* < 0.001) and explained 34.6% (Nagelkerke R2) of the variance in illicit drug use during the past 12 months. The model correctly classified 82.4% of the sample. Of the 1371 participants, 49 (3.6%) were excluded from the regression analysis due to missing data. Of the remaining 1322, 769 (60%) had used illicit drugs during the last 12 months. 

The strongest significant association with illicit drug use during the last 12 months was found for having ever been to an illegal festival (OR, 4.8; CI, 3.6 to 6.4) (Table 5). An increased likelihood of having used illicit drugs during the past 12 months was associated with: having used tobacco at least fortnightly (OR, 2.5; CI, 1.8 to 3.4), having ever used tobacco (OR, 2.1; CI, 1.5 to 3.1), and drinking alcohol at least fortnightly (OR, 1.5; CI, 1.0 to 2.0). The following motives for going out increased the likelihood of illicit drug use during the last 12 months: ‘to seek excitement’ (OR, 2.0; CI, 1.5 to 2.7); ‘to escape daily life’ (OR, 1.5; CI, 1.1 to 1.9); and ‘to explore one’s mind’ (OR, 1.4; CI, 1.0 to 1.9). In contrast, the motive ‘to see an artist’ decreased the likelihood of having used illicit drugs during the past 12 months (OR, 0.6; CI, 0.5 to 0.9). There were no significant associations for the remaining factors (Table 5).

### 3.5. Consequences of Drug Use

The vast majority of respondents stated that they frequently had experienced various positive consequences after drug use (Table 6). Positive experiences ranged from effects on perception (perception of music, expanded consciousness, sense of enlightenment) to emotional and social effects (intense pleasure, reduced inhibitions, closeness to others, feelings of love and empathy, making friends). However, a large proportion of respondents stated that they had experienced negative consequences, albeit less frequently than positive consequences, such as mood disturbances related to illicit drug use, mainly low mood or anxiety in the days immediately following drug use, agitation, and panic attacks or anxiety (Table 6). Both memory loss and sleep disturbances were also common negative experiences after illicit drug use. Several illicit drug users experienced physical symptoms, including vomiting, palpitations, and overheating. More severe physical symptoms experienced by fewer respondents included breathing difficulties, fainting or collapsing, and an inability to move. The respondents experienced mental health problems at a higher frequency than physical health problems (Table 6). Furthermore, 60% of respondents experienced unexpected effects of the drug, indicating that they might unknowingly have consumed another substance than intended or that they lacked knowledge about the drug’s effect or the effects of polydrug use.

### 3.6. The Drug Use Disorder Identification Test (DUDIT)

Participants who had used any illicit drug during the past 12 months also filled out the DUDIT questionnaire (Table 7). Every third respondent reported using drugs two to four times a month or more often. One in four persons reported use of drugs at least three to four times during a typical drug-taking day. One in ten used several types of drugs on the same occasion (polydrug use) at least two to four times a month. About one in four reported experiencing strong craving that could not be resisted, the majority of which had experienced this less than monthly. Similarly, about one in six and one in seven participants reported an inability to stop using drugs when having started or using drugs the morning after drug use, respectively. About one in three respondents had neglected to do something they should have done, and almost half of the respondents reported having had feelings of guilt or bad conscience related to illicit drug use (Table 7). 

Based on the sum of the DUDIT scores for each item, a composite score was calculated (possible range: 0–44). The number and frequency of drug users (*n* = 889) who exceeded the recommended cut-off (2 for women, 6 for men, reflecting population averages) was 67.5% (*n* = 600). Furthermore, 2.5% (*n* = 22) exceeded the recommended cut-off (25) for a diagnosis of substance use disorder. Most participants reported cannabis as the main drug, followed by ecstasy, amphetamine, and cocaine (Table 8). The DUDIT score among participants stating these drugs were (median and interquartile range): cannabis, 7 (3–11); ecstasy, 6 (4–8); amphetamine, 8 (5–11); cocaine, 8 (4.5–11.5); indicating no major difference between users of these drugs regarding their abuse. In contrast, although only six participants stated the use of heroin as the main drug, the median score was 25.5 (16.5–34.5 interquartile range), indicating dependence. 

To assess general well-being during the last two weeks, participants answered the WHO positive well-being questions (1–5) regarding being in good spirits, being active and vigorous, feeling calm and relaxed, feeling fresh and rested, as well as being interested in things. The standardized composite score (0–100) obtained from these questions was significantly different across drug frequency categories as assessed by the DUDIT-1 question regarding drug use frequency during the last 12 months (Kruskal–Wallis, *p* = 0.041). The WHO composite score was 68 among those who were not shown the DUDIT questionnaire since they had not stated drug use during the last 12 months (IQR: 52–76, *n* = 482), as well as among those stating ‘never’ (IQR: 48–76, *n* = 119) or ‘once a month or less often’ (IQR: 52–76, *n* = 467) in the DUDIT-1 question. The composite score among participants who had stated drug use ‘two to four times a month’ was 64 (IQR: 52–80, *n* = 169), among those stating ‘two to three times a week’ was 60 (IQR: 36–69, *n* = 54) and among those stating ‘four times a week or more often’ was 64 (40–76, *n* = 80). 

## 4. Discussion

The present study demonstrates that illicit drug use is prevalent among young adults regularly attending EDM nightlife events in Sweden. In addition, patterns of drug use, factors influencing drug use, as well as positive and negative consequences after using drugs are presented.

### 4.1. Prevalence of Illicit Drug Use and Settings Where Drugs Are Used

Participants had visited a median number of 15 EDM events (IQR 10–25) during the past 12 months, indicating frequent nightlife participation. A majority (72%) of the participants had used illicit drugs during their lifetime, and 59% reported having used them during the past year. The most commonly used drug was cannabis, followed by ecstasy, cocaine, and amphetamine. Compared with young adults in the general population in Sweden, last year use of these drugs, was between 5 and 20 times more prevalent among our study sample [10].

Compared with previous studies conducted in nightlife settings, the present study showed a similarly high prevalence of illicit drug use during the previous year as that among club goers in Norway (43%) [20] and among staff at licensed premises in Stockholm (47% among 18- to 24-year-olds) [56]. Furthermore, in line with previous studies, the most commonly used drugs were cannabis, followed by ecstasy, cocaine, and amphetamine [4,11,12,13,15,17,20,23,24,25,26,27,41]. Moreover, 11% of participants reported last-year use of ketamine. A recent study among attendees of EDM events, nightclubs and festivals in New York showed that last-year use of ketamine had increased from 6% to 15% between 2016 and 2019 [16]. Additionally, in the New York study, last-year use of central stimulants (data of 2017) was less prevalent than in the present study [16].

The present results clearly indicate that nightclubs and festivals are the main settings for the use of ecstasy, cocaine, and amphetamine, confirming previous findings of biological drug toxicology tests in these settings [18,21,22]. Cannabis was most often consumed in the home. Mushrooms and LSD were also mostly used at home, while ketamine was reportedly used at home, as well as at illegal and legal festivals.

### 4.2. Factors Associated with Drug Use

Illicit drug use was common in all age groups but most prevalent among 22- to 28-year-olds. This is in line with previous research conducted in the nightlife setting among Swedish staff at licensed premises and among club goers in Norway, where past year drug use was higher among 18- to 25-year-olds than older age groups [20,56]. In addition, a Belgian study reported that last-year drug use was lower in nightlife goers above 26 years of age, which was suggested to be due to increased life responsibilities [52]. Hence, the inverted U-shape seen here might be related to the age of onset for most drugs in the youngest age group, as well as to increased responsibilities in older age groups. Similar to other studies, the current results confirm that drug use starts in and is high in young adulthood. Age of onset was about the same for ecstasy, cocaine, and amphetamine (i.e., 21 years), lower for cannabis (17 years), and higher for ketamine (23 years). 

The use of central stimulants was more prevalent among women than men in the current study. However, the majority of participants were men, a potential recruitment bias that could hinder the investigation of gender effects. While some nightlife studies have found higher illicit drug use prevalence among men compared with women [13,40,52], previous Swedish nightlife studies showed no statistically significant gender differences [17,18,56]. 

The frequency of alcohol and tobacco use was correlated with the number of illicit drugs taken during the past 12 months and using alcohol or tobacco at least every other week increased the likelihood of use of illicit drugs. The present results thus add to the existing evidence that—instead of substituting each other—drinking hazardous amounts of alcohol and taking illicit drugs often coexists among nightlife attendees [18,20,37,40,42]. 

In addition to alcohol and tobacco use frequency, the factors associated with illicit drug use were having visited an illegal festival and the motives for going out ‘to seek excitement’ and ‘to escape daily life’. This finding is in line with previous studies correlating sensation-seeking traits with illicit drug consumption [57,58,59]. In contrast, the motive to ‘see an artist’ reduces the likelihood of using illicit drugs. Furthermore, most people reported that they go out to ‘have fun’ and ‘listen to music’ and ‘to dance’. In contrast, few people stated that drug taking is important in going out. Together, these results indicate that the nightlife experience is not necessarily related to drug use and that music culture should be fostered to make drug use less likely and perhaps less necessary. 

### 4.3. Consequences

Participants mainly experienced positive consequences after drug use, including effects on perception, as well as emotional and social effects, illustrating the motivation for their use. For example, the vast majority of participants reported frequent experiences of ‘enhanced perception and enjoyment of music’, which could explain the high prevalence of drug use at nightclubs and EDM events [13,26,41,60,61]. Compared with positive experiences, negative ones were generally experienced less often, although also fewer participants chose to answer these questions. Therefore, this difference could have been exacerbated by reporting bias, where participants are more willing to illuminate positive aspects of drug use. The most prevalent negative consequences from illicit drug use reported by participants in the present study were related to mental health, but physical manifestations were also reported, symptoms ranging from palpitations and overheating to breathing difficulties, paralysis, and collapsing. The most common mental health consequences were mood disturbances (e.g., dysphoria), anxiety, panic attacks, sleep disturbances, and memory loss. Previous studies have attributed such consequences to the use of cannabis, ecstasy, amphetamine, and cocaine [5,29,33,34,35,36,62,63,64], which are also the drugs most frequently used by participants in the present study. A recent meta-analysis shows that ecstasy users display deficits in the serotonergic transporter in several brain areas. However, mostly heavy ecstasy use was present in the studies included and the effects of low and moderate use remain largely unknown [65]. The present results confirm negative experiences reported previously in three nightlife studies [4,45,51] and further strengthen the finding of a recent study that adverse consequences of drug use occur among nightlife participants [44]. Furthermore, although the majority of the illicit drug users in the current sample did not suffer from substance use disorder (according to the cut-off of 25 in the DUDIT), one in four and one in six persons reported having experienced strong craving and inability to stop taking the drug when initiated. Furthermore, participants stating drug use two to four times a month or more often reported lower general well-being (WHO composition score) compared with those not using or using drugs less often. From the present results, it cannot be determined whether illicit drug use decreases general well-being, if a lower general well-being increases the likelihood of drug use, or if both have common underlying factors. Nevertheless, the findings on general well-being as well as reported negative experiences after use of illicit drugs hint to a negative effect of drug use on the mental health of EDM event goers and that if drug use persists or exacerbates, they could be at increased risk of future development of mental disorders. 

Although the effects of illicit drugs on mental and physical health are well known, they have mostly been studied in dependent patients and in recreational drug users [5,34,46,47,48,49,50]. In the present study, we could show that EDM nightlife goers experience mostly positive effects in the majority of the time. However, a number of adverse consequences have also been reported, despite the infrequent use of most drugs except cannabis. In line with previous research, participants also reported consequences such as sexual activity one later regrets, driving under drug-influence, as well as aggression [66,67,68].

### 4.4. Implications for Prevention

The present results indicate the need and possibility for prevention interventions at EDM events. Regarding such interventions, nightlife settings are a suitable arena for reaching young adults of various socioeconomic statuses, including students, employed, and unemployed persons. In 2001, our research group, STAD, developed and implemented a multi-component community-based program for preventing drug use in the nightlife scene called the ‘Clubs Against Drugs’ [17,69]. The program was developed in co-production with different stakeholders, such as authorities, club owners, and the police and consisted of different components, including training, enforcement, PR, and media advocacy. Clubs Against Drugs was based on a similar program that had been developed to reduce alcohol use and associated problems at licensed premises (Responsible Beverage Service, RBS) [70,71,72,73,74]. Studies have shown that the implementation of the Clubs Against Drugs program resulted in a decrease in illicit drug use among nightlife attendees and staff [17,69]. STAD’s RBS program has continued and has been disseminated in Sweden [75], while the activities in the Clubs Against Drugs program have decreased due to lack of funding. A follow-up study conducted in 2016/17 among staff at licensed premises in Stockholm indicated that illicit drug use had increased since the last follow-up in 2007/08, demonstrating a need for preventive interventions in the nightlife scene [56]. The present results could guide the different stakeholders in co-production of a new program. For example, the acute physical symptoms should guide harm reduction approaches, such as training of nightlife staff and access to medical emergency services. Another example could be traffic controls regarding alcohol and drug use around the event. In addition, considering the effects of sexual risk taking, discussions on the topic of sexual consent should include drug use.

Nevertheless, the present results also demonstrate that we need to find new ways of reaching the target group. A large number of young adults consume cannabis, mainly at home, and the majority had started to use it before they reached the age limit to visit licensed premises. Research suggests that brief or digital interventions based on motivational interviewing and personal feedback can reduce risky behaviors, drug use, and related consequences [76,77,78,79,80].

### 4.5. Strengths and Limitations

The strengths of the present study are that it consisted of an in-depth analysis of drug use patterns and a large sample of frequent EDM event visitors. Furthermore, there was no internal drop-out regarding drug use prevalence and frequency among the survey completers. Analyses showed that drug use was not more prevalent among the study population than among persons excluded from analysis. A limitation of the study is the substantial internal drop-out regarding questions of main setting of use and consequences of drug use. Moreover, 72% of the respondents were male, which might reflect a selection bias, as indicated by the following studies: (1) offline studies at one large festival and a large EDM event in Sweden consisted of 64 percent males in each study [18,81]; (2) a comparison of online and offline recruitment in the remaining European countries of the ALAMA study indicates that the online and offline sample consisted of 69% and 58% males, respectively [82]. Furthermore, the online participants were on average one year younger compared with offline participants, and drug use was slightly less prevalent in the online sample [82]. Another limitation is that regarding past 12-month drug use, it is unclear the extent to which this occurred in nightlife settings. Furthermore, the analyses in the current study were largely exploratory, and multiple comparisons were not adjusted for.

## 5. Conclusions

Illicit drug use is prevalent among persons who frequently attend Swedish EDM events. Attendees report a variety of negative consequences on mental and physical health related to their illicit drug use. Furthermore, frequent alcohol or tobacco use is associated with illicit drug use. Therefore, there is a need and a possibility to use the nightlife scene as a strategic arena for preventive interventions addressing both illicit drug and alcohol use. Knowledge on drug use prevalence, patterns, and negative consequences can be used to motivate stakeholders to consider preventive interventions and could help identify strategies needed to reduce drug use and associated problems among nightlife attendees. Specifically, the use of the central stimulants, ecstasy, cocaine, and amphetamine, but also of ketamine, could be prevented in nightlife venues. In contrast, cannabis use is most prevalent at home and might need interventions such as internet-based programs.

## Figures and Tables

**Table 1:** Demographic characteristics of the participants (*n* = 1371).

Demographic Characteristics	% (*n*)
Gender	
Male	72.3 (991)
Female	25.9 (355)
Other	1.8 (25)
Occupation ^1,4^	
Full time work	44.7 (613)
Part time work	18.0 (247)
Student	37.2 (510)
Neither education, employment, nor training	5.7 (78)
Urbanicity ^1^	
Large town/city	60.8 (834)
Small to mid-sized town	29.6 (406)
Rural/countryside	7.9 (108)
Education ^2,4^	
Completed primary school	97.2 (1333)
Currently attending high school	6.7 (92)
Completed high school	90.7 (1244)
Currently attending university	29.0 (397)
Completed university degree	23.0 (316)
Relationship status ^1^	
Single	51.7 (709)
Married or in a civil partnership	3.3 (45)
Divorced or separated	0.5 (7)
In a relationship, not living with partner	17.7 (242)
In a relationship and living with partner	25.2 (345)
Sexual orientation ^3^	
Men attracted to women	67.5 (926)
Women attracted to men	19.5 (268)
Women attracted equally to men and women	3.7 (51)
Men attracted to men	2.3 (31)
Women attracted to women	1.1 (15)
Men attracted equally to men and women	0.9 (12)
Women not attracted to either men or women	0.1 (2)

^1–3^ Data were missing for ^1^ *n* = 23, ^2^ *n* = 24, ^3^ *n* = 66. ^4^ The subcategories were asked for separately and are not mutually exclusive.

**Table 2:** Prevalence of illicit drugs less commonly used (*n* = 1371).

Substance	Lifetime % (*n*)	Last 12 Months % (*n*)
Synthetic Cannabinoids	16.0 (219)	1.1 (15)
Synthetic Hallucinogens	14.1 (193)	5.3 (73)
Prescription Opioids	13.9 (190)	7.1 (97)
DMT ^1^	8.8 (120)	3.9 (53)
Mephedrone	5.0 (68)	0.5 (7)
GHB ^2^	3.4 (47)	1.5 (21)
Synthetic Dissociatives	3.3 (45)	0.7 (10)
4-FA/4-FMP ^3^	2.0 (27)	1.0 (14)
Heroin	1.5 (20)	0.4 (5)

^1^ N,N-Dimethyltryptamine, ^2^ Gamma-hydroxybutyrate, ^3^ 4‑Fluoramphetamine.

**Table 3:** Main setting of illicit drug use stated by drug users (% of respondents).

Substance (*n* Respondents)	Night- Clubs	Pub/ Bars	Licensed Festivals	Illegal Festivals	Public Spaces	House Party ^1^	At Home ^2^
Cannabis (692) ^3^	0.6	0.7	3.2	3.2	8.5	14.9	65.0
Ecstasy (498) ^4^	19.1	0.2	26.9	35.1	0.2	8.6	8.4
Cocaine (380) ^5^	38.9	7.6	7.6	10.3	-	24.5	9.2
Amphetamine (311) ^6^	17.4	3.9	12.2	29.6	1.9	11.3	15.1
LSD (214) ^7^	2.8	-	10.7	8.9	10.7	2.3	60.3
Mushrooms (173) ^8^	1.2	0.6	8.1	6.4	18.5	9.2	49.7
Ketamine (149)	13.4	1.3	20.1	24.2	1.3	16.8	22.8

^1^ A party at someone’s home. ^2^ At the participant’s own home or a friend’s home. The remaining respondents stated ‘other’ as the main setting of use: ^3^ 3.9%, ^4^ 1.4%, ^5^ 1.8%, ^6^ 8.7%, ^7^ 4.2%, ^8^ 6.4%. LSD, lysergic acid diethylamide.

**Table 4:** Use of illicit drugs during the last 12 months across gender.

Drug Use during the Last 12 Months	Males % (*n*)	Females % (*n*)	X^2^ (df = 1), *p*-Value
Any illicit drug	56.4 (559)	64.8 (230)	7.57, 0.006
Cannabis	49.8 (494)	53.5 (190)	1.41, 0.235
Ecstasy	34.7 (344)	42.5 (151)	6.88, 0.009
Cocaine	26.2 (260)	32.7 (116)	5.39, 0.020
Amphetamine	21.3 (211)	26.8 (95)	4.45, 0.035
LSD	15.4 (153)	15.5 (55)	0.00, 0.981
Magic Mushrooms	12.4 (123)	12.7 (45)	0.02, 0.897
Ketamine	10.0 (99)	13.0 (46)	2.40, 0.122

LSD—lysergic acid diethylamide.

**Table 5:** Binomial logistic regression of illicit drug use during the last 12 months. Due to missing data (*n* = 49), 1322 were included in the analysis. All variables are dichotomous or were dichotomized, except for the continuous variable age and for the variable higher education ^1^.

Factors	Wald	OR	95% CI	*p*-Value
Age	3.00	0.97	0.94–1.00	0.083
Gender	3.05	0.76	0.57–1.03	0.081
Occupation				
Full time work	0.02	1.03	0.66–1.61	0.902
Part time work	2.02	1.36	0.89–2.09	0.155
Student	0.10	0.93	0.59–1.48	0.757
Higher education ^1^		1.31		0.519
Number of events attended ^2^	0.14	1.00	1.00–1.01	0.705
**Ever visited an illegal festival**	**110.29**	**4.77**	**3.56–6.38**	**<0.001**
Visited club ≥ monthly ^2^	0.96	0.86	0.63–1.17	0.328
Visited legal festival ≥ monthly ^2^	0.65	1.17	0.80–1.70	0.420
Visited pub ≥ monthly ^2^	2.12	1.29	0.92–1.82	0.146
Visited house party ≥ monthly ^2^	0.24	0.93	0.71–1.23	0.626
**Tobacco consumption ever**	**14.92**	**2.14**	**1.45–3.14**	**<0.001**
**Alcohol at least fortnightly ^2^**	**5.19**	**1.48**	**1.06–2.08**	**0.023**
**Tobacco at least fortnightly ^2^**	**37.79**	**2.52**	**1.88–3.38**	**<0.001**
Motives to go out ^3^:				
Friends are going	0.03	0.97	0.70–1.36	0.875
To meet new people	0.83	0.88	0.67–1.16	0.363
**To seek excitement**	**21.50**	**1.99**	**1.49–2.67**	**<0.001**
To open up to friends	0.05	1.03	0.78–1.36	0.824
To drink alcohol	0.02	0.98	0.73–1.31	0.889
**To escape daily life**	**7.16**	**1.46**	**1.11–1.93**	**0.007**
**To explore mind**	**4.25**	**1.38**	**1.02–1.87**	**0.039**
To cope with problems	0.84	0.87	0.64–1.18	0.361
**To see an artist**	**7.06**	**0.64**	**0.46–0.89**	**0.008**

^1^ Categories: completed, currently attending, never started, ^2^ during the last 12 months, ^3^ the variable was dichotomized by combining ‘slightly important’ and ‘very important’ (yes) ‘not important’ and ‘not very important (no). Significant factors were highlighted in bold for clarity.

**Table 6:** Experienced consequences of the use of illicit drugs. Response options were from 0 (never) to 10 (every time). The number of respondents is shown for each experience. Data are presented as percentage of respondents who gave a response between 1 and 10. Hence, the remaining respondents answered ‘never’, i.e., had not had this experience. The median and interquartile range of those that had responded between 1 and 10 is also provided.

Experienced Consequences (*n* Respondents)	Percentage of Respondents	Median (Interquartile Range)
Enhanced perception, enjoyment of music (755)	95.4	8 (6–10)
Closeness to others (716)	95.0	7 (5–9)
Feelings of love and empathy (747)	94.9	7 (5–9)
Intense pleasure (741)	94.6	7 (5–9)
Making new friends (704)	92.0	6 (4–8)
Expanded consciousness (693)	91.5	6 (4–8)
Reduced inhibitions (671)	91.1	5 (3–7)
Increased sense of enlightenment (652)	90.2	6 (3–8)
Low mood or anxiety in days afterwards (564)	77.8	3 (2–5)
Memory loss (570)	76.1	3 (2–5)
Agitation (530)	74.2	3 (2–5)
Spending money you cannot afford to (481)	64.7	3 (2–5)
Problems with sleep in days after use (443)	60.7	2 (1–5)
Effect of the drug not as expected (417)	60.0	2 (1–3)
Vomiting (464)	58.8	2 (1–3)
Palpitations (436)	58.5	2 (1–4)
Overheating (421)	58.4	2 (1–4)
Panic attacks or anxiety (452)	57.1	2 (1–3.25)
Driven/been driven by someone under the influence of alcohol/drugs (373)	45.0	2 (1–3)
Arguments with friends (367)	44.7	1 (1–3)
Sexual activity you later regret (367)	40.3	2 (1–4)
Aggression/victim of aggression (372)	39.8	1 (1–3)
Accidents (374)	38.2	1 (1–2)
Missing work or important commitments (361)	38.2	2 (1–4)
Breathing difficulties (346)	31.5	1 (1–3)
Legal problems (e.g., being arrested) (320)	27.8	2 (1–4)
Problems with a bouncer (314)	27.4	1 (1–4)
Inability to move (298)	24.8	1.5 (1–3)
Fainting or collapsing (310)	20.0	1 (1–2)
Seeking or receiving emergency medical treatment (287)	15.3	1 (1–3)

**Table 7:** DUDIT (Drug Use Disorder Identification Test) among drug users. Participants who had stated drug use during the last 12 months were asked to fill out the DUDIT. Data are presented as percentage of participants (*n* = 889). There were no missing data. Questions relate to illicit drug use.

**DUDIT 1–2**	**Never**	**≤Monthly**	**2–4 Times a Month**	**2–3 Times a Week**	**≥4 Times a Week**
Drug use ^1^ frequency	13.4	52.5	19.0	6.1	9.0
Polydrug use ^2^	40.7	46.7	10.1	1.6	0.9
**DUDIT 3**	**None**	**1–2 Times**	**3–4 Times**	**5–6 Times**	**≥7 Times**
Times drug taking ^3^	12.4	65.8	15.4	4.3	2.1
**DUDIT 4–** **9**	**Never**	**<Monthly**	**Monthly**	**Weekly**	**Daily**
Heavily influenced	27.1	54.6	12.7	4.2	1.5
Strong craving that cannot be resisted	75.5	16.9	4.5	1.6	1.6
Unable to stop once started ^4^	82.7	11.9	3.4	0.9	1.1
Neglected to do something ^4^	61.1	29.2	6.4	2.7	0.6
Drug morning after use ^4^	84.5	10.5	3.1	1.1	0.8
Guilt, bad conscience ^4^	51.6	37.2	6.6	3.1	1.3
**DUDIT 10–** **11**	**No**	**Yes, but not over the Past Year**		**Yes, over the Past Year**	
Hurt ^5^ yourself or others	77.4	14.6		8.0	
Advice to stop ^6^	67.9	19.0		13.0	

^1^ Other than alcohol or tobacco, ^2^ using more than one type of drug on the same occasion, ^3^ on a typical day when drugs are taken, ^4^ over the past year, ^5^ mentally or physically, ^6^ worry about drug use expressed by friend, doctor, nurse, or anyone else.

**Table 8:** Main drugs reported on the DUDIT questionnaire.

DUDIT Drugs	% (*n*)
No response	6 (53)
Cannabis	43.6 (388)
Ecstasy	15.9 (141)
Alcohol ^1^	11.0 (98)
Amphetamine	7.1 (63)
Cocaine	7.0 (62)
Nitrous oxide	2.2 (20)
Tobacco ^1^	1.6 (14)
LSD	1.2 (11)
Prescription Opioids	0.8 (7)
Heroin/Opiates	0.7 (6)
Ketamine	0.6 (5)
Magic Mushrooms	0.3 (3)
Benzodiazepines	0.3 (3)
Others ^2^	1.7 (15)

^1^ Observe that the questionnaire clearly states ‘drugs other than alcohol or tobacco’, ^2^ not specifically stated or no psychoactive substance (*n* = 4), pregabalin (*n* = 3), methylphenidate (*n* = 2), d-lysergic acid amide (LSA) (*n* = 1), new psychoactive substances (*n* = 1), gamma-hydroxy-butyrate/GHB (*n* = 1), topiramate (*n* = 1), and coffee (*n* = 1). LSD, lysergic acid diethylamide.

## Data Availability

The data presented in this study are available on request from the corresponding author. The data are not publicly available due to ethical reasons.

## Supplementary Materials

The following materials are available online at https://www.mdpi.com/article/10.3390/ijerph18094789/s1, Table S1: Source of information regarding the survey, Table S2: Comparison of demographics and drug use prevalence between participants included and excluded from analysis, Table S3: Frequency of music styles played at EDM venues attended, Table S4: Motives to go out rated for their importance, Table S5: Venues attended during lifetime, the past 12 months, as well as age of onset.

## Appendix A

To identify potential factors that could predict use of illicit drug use during the last 12 months, a binary logistic regression was performed. In general, factors clearly being associated with drug consumption, such as experienced consequences of drug intake, were not included. To avoid overfitting, the number of independent variables was reduced by excluding variables with infrequent categories (below 15%) or where the response rate was below 50%. Therefore, the following variables were excluded: music genres being played at the EDM events, certain motives for going out (to have sex, to look for partner, to take drugs, to have fun, to listen to music; see supplementary Table S4), having ever been to a club/pub/legal festival (see, supplementary Table S5), having ever consumed alcohol, having consumed alcohol or smoked tobacco during the last 12 months, urbanicity, neither working nor studying, all education levels except for higher education, sexual orientation, relationship status, number of types of venues (1–5) attended during lifetime or during the last 12 months (Table S5), number of friends taking drugs, and number of friends who attended six or more electronic dance events during the last 12 months. The other factors were recoded into fewer categories to increase frequencies: regarding motives categories ‘very important’ and ‘slightly important’, as well as ‘not important’ and ‘not very important’ were collapsed; visiting frequencies of different types of venues were collapsed to ‘monthly or more often’ and ‘less than monthly’; and frequency of tobacco or alcohol consumption was collapsed to ‘fortnightly or more often’ and ‘less than fortnightly’; the gender ‘other’ was excluded. For the variables remaining in the analysis, multicollinearity testing (using linear regression where every variable was once tested as a dependent variable against the other variables) showed that none of the variables had a variance inflation factor (VIF) above 3, i.e., no variable correlated with the other variables.
