# Supplementary material for: Illicit Drug Use and Associated Problems in the Nightlife Scene: A Potential Setting for Prevention
Source: Int J Environ Res Public Health. 2021 Apr 30;18(9):4789. doi: 10.3390/ijerph18094789 (PMC8125188; doi:10.3390/ijerph18094789)
Supplement: Supplementary file 1 [file ijerph-18-04789-s001.zip › ijerph-1173066-supplementary.pdf]

Table S1. Source of information regarding the survey

Event Info	Percent
Facebook	87.2
Friends	84.5
Word of Mouth	56.8
E-mail list or newsletter	42.5
Offline advertising	26.7
Other websites	23.9
Resident Advisor	16.5
Twitter	3.8
Other	3.3
Pirate or online radio stations	3.2

Participants (n=1371, no missing data) stated where they got information about the survey.

Table S2. Comparison of demographics and drug use prevalence between participants included and excluded from analysis

	A) included % (n)	B) excluded % (n)	C) excluded % (n)
Gender			
Male	72.3 (991)	72.6 (454)	74.2 (743)
Female	25.9 (355)	25.9 (162)	24.0 (240)
Other	1.8 (25)	1.4 (9)	1.9 (19)
Age in years			
Mean ± SD	24.8 ± 4.5	25.4 ± 5.0	24.9 ± 5.0
Min-max	18-34	18-51	14-51
Illicit drugs ever	71.9 (986)	83.0 (519)	-
Ever use of			
Cannabis	68.2 (935)	79.8 (499)	-
Ecstasy	47.8 (656)	64.8 (405)	-
Cocaine	37.9 (519)	56.3 (352)	-
Amphetamine	35.4 (485)	51.5 (322)	-
Use last 12 months of			
Cannabis	51.0 (699)	37.0 (231)*	-
Ecstasy	36.5 (500)	25.6 (160)**	-
Cocaine	27.9 (382)	18.2 (114)***	-
Amphetamine	22.9 (314)	13.9 (87)****	-

a) Included in the study were 1371 participants who completed the survey and not indicating use of "spanglers", as well as met the following inclusion criteria: age of 18-34 years and having visited at least six electronic dance music events during the previous 12 months.
b) A group excluded from analysis (n=625) due to not completing the survey (n=498), stating the use of "spanglers" (n=4) or not meeting the inclusion criteria (see a, n=123) but still answering relevant questions on drug use. Percentages of internal dropout: *31.0%, **35.2%, ***30.2%, ***29.6%.
c) remaining group (n=1002) excluded from analysis had not completed the survey and stopped answering the questions before drug use (median time: 4 min). Whereas at earlier questions (e.g. club ever attendance or motive to go out) 302 individuals had not answered (probably stopped the survey), at the question before drug use "events info" (Where they got to know about the survey) 958 people had not answered any of those questions.

Table S3. Frequency of music styles played at EDM venues attended

	respondents % (n)	Yes (% of respondents (n))	median (IQ range)
House	93.3 (1279)	98.5 (1260)	7 (5-8)
Techno	86.9 (1191)	97.6 (1162)	6 (3-8)
Electro	72.9 (999)	94.1 (940)	4 (2-7)
Hip-hop	67.6 (927)	91.3 (846)	4 (2-7)
Trance	67.5 (926)	99.0 (917)	3 (1-5)
Disco	62.4 (856)	88.1 (754)	3 (1-5)
Dubstep	60.3 (827)	84.4 (698)	2 (1-3)
DnB	60.3 (827)	89.0 (736)	2 (1-4)
RnB	60.2 (825)	88.4 (729)	4 (1-6)
Goa	58.1 (796)	86.4 (688)	3 (1-6)
Reggae	54.3 (745)	85.9 (640)	2 (1-4)
HardD	53.0 (727)	78.7 (572)	2 (1-4)
IDM	47.5 (651)	79.1 (515)	2 (1-5)
Hardcore	45.2 (620)	73.4 (455)	1 (0-4)
New Wave	38.4 (527)	67.7 (357)	1 (0-4)
Other	49.0 (672)	47.3 (318)	0 (0-5)

Of 1371 participants. response frequency varied for each music style (left column). Percent of respondents stating that a certain music style was played at the EDM events that they attend. as well as median frequency the music style was played from 0 (never) to 10 (all the time) are displayed. Other genres that participants reported were: Acid. Ambient, Balearic, Breakcor, Commerci, Psy, EBM, Funk, Future B, Hardstyle, Indie, minimal, Pop, Psybient, Progress, Reagaeto, Rock, slow tec, soft house, Synth, Trap, triphop, Industri, etc.

Table S4. Motives to go out rated for their importance

Motives	Not important	Not very important	Slightly important	Very important
To have fun	0.1	0.7	9.3	89.8
To listen to music	0.4	2.6	19.9	77.1
To dance	2.5	9.8	27.6	60.1
My friends are going	3.2	17.4	44.8	34.6
To see a particular artist or event	4.2	16.4	46.4	33.0
To seek excitement	15.4	27.3	39.6	17.7
To open up to my friends	23.2	28.3	34.1	14.4
To explore my mind	24.9	36.3	24.4	14.4
To escape my daily life	24.4	27.9	34.1	13.6
To meet new people	10.9	40.5	38.1	10.5
To drink alcohol	21.7	33.0	35.2	10.1
To cope with my problems	37.6	29.5	25.0	8.0
To look for sex	61.1	29.0	8.8	1.2
To take drugs	57.0	28.2	13.1	1.6
To look for a partner	67.2	26.1	6.1	0.6

Participants (n=1371, no missing data) rated the importance of different motives to go out.

Table S5. Venues attended during life-time, the past 12 months, as well as age of onset

Venue	Ever<br>% (n)	12 months<br>% (n)	≥ monthly<br>% (n)	Age first<br>median
Club	96.2 (1319)	95.3 (1307)	67.0 (918)	18
Legal festival	95.3 (1306)	89.9 (1232)	16.3 (224)	18
House party	93.8 (1286)	91.2 (1250)	46.2 (633)	15
Pub	92.2 (1264)	91.4 (1253)	73.1 (1002)	18
Illegal festival	67.7 (928)	57.5 (789)	22.0 (302)	20

Participants stated which types of venues they had attended during their life-time and during the past 12 months. There was no missing data for "ever"-attendance. The remaining questions were only shown to participants who had stated "ever-attendance". Percentages are given of all participants (n=1371). From the question of how frequently these venues were attended during the last 12 months, participants who stated at least monthly attendance (or more often) are presented. A house party refers to a private party at someone's home (not the music style).
